# Mechanical Behaviour of Green Epoxy Composites Reinforced with Sheep and Dog Wool from Serra Da Estrela

**DOI:** 10.3390/polym16223115

**Published:** 2024-11-07

**Authors:** Cláudia Antunes, Ana Paula Costa, André Costa Vieira, Joana Costa Vieira

**Affiliations:** 1 Fiber Materials and Environmental Technologies Research Unit (FibEnTech-UBI), University of Beira Interior, Rua Marquês D’Ávila e Bolama, 6201-001 Covilhã, Portugal; claudia.sb.antunes@ubi.pt (C.A.); anacosta@ubi.pt (A.P.C.); 2 Center for Mechanical and Aerospace Science and Technologies (C-MAST-UBI), University of Beira Interior, Rua Marquês D’Ávila e Bolama, 6201-001 Covilhã, Portugal; andre.costa.vieira@ubi.pt

**Keywords:** animal fibres, dog wool fibre, sheep wool fibre, green composites, mechanical properties

## Abstract

Environmental awareness has led industries and consumers to replace products derived from oil resources with products derived from natural sources. In the case of the composite materials industry, the replacement of synthetic fibres with natural fibres has increased in recent years. To study the influence that different types of natural fibres and different textile manufacturing techniques have on the mechanical properties of composites, bio-based epoxy matrix composites reinforced with different natural animal fibres were produced, some reinforced with sheep’s wool and others with dog wool, which were later subjected to bending and tensile tests. From the authors’ knowledge, there are few studies of composites produced with animal fibres, and even fewer with dog hair. The textile structures used as reinforcement were created using crochet, knitting, and weaving techniques. Prior to the composites production, the fibres were characterized by X-ray Diffraction (X-RD), and the yarns produced from these fibres were subjected to tensile tests. The results obtained suggest that the number of yarns and the diameter of the needles used during the production of the reinforcement have a significant impact on the mechanical properties of the composites. The green epoxy resin composites reinforced with sheep’s wool exhibit higher values of flexural strength, tensile strength, and Young’s modulus than those reinforced with dog wool, with average increases of 36.97%, 45.16%, and 72.99%, respectively. It was also possible to verify that the composites reinforced with woven fabrics and crocheted fabrics exhibit the highest values of tensile strength, flexural strength, and Young’s modulus. Additionally, the composites reinforced with woven fabrics exhibit the highest values of deformation at first failure/break and toughness.

## 1. Introduction

Due to new environmental regulations and the depletion of oil resources, along with their high costs and adverse environmental impacts, the use of natural resources has increased, which has led industries and researchers to search for and develop more sustainable materials. An example of such materials is the polymer composites reinforced with natural fibres [1,2,3].

Natural fibres can be classified into three types [4,5]:

- Vegetal fibres, which are obtained from various parts of plants (e.g., Ramie, Flax, Hemp, cotton)
- Animal fibres, which are derived from animal hair and secretions (e.g., wool, feathers, silk)
- Mineral fibres, which come from inorganic natural resources (e.g., asbestos)

Natural fibres are low cost, highly available, and environmentally friendly, with relatively low density, high impact resistance, and high flexibility. Additionally, their processing methods are simple, environmentally friendly, and less abrasive for equipment. They also have good mechanical properties compared to those of synthetic fibres [3,6,7,8,9,10,11].

However, natural fibres have some downsides. For instance, their mechanical and physical properties exhibit low consistency, and they have a high moisture absorption capacity (hygroscopicity). Additionally, they are flammable, susceptible to degradation by microorganisms and sunlight, and require lower processing temperatures [3,12].

Table 1 highlights the properties of some natural and synthetic fibres [4,13,14].

In the field of natural fibres, vegetal fibres have dominated the industry. However, the use of animal fibres has grown significantly in recent years. Besides the previously mentioned advantages, their use helps reduce solid waste that would otherwise end up in landfills [13].

Animal fibres are made up of keratin. Keratin is a fibrous structural protein rich in amino acids that can be found in various parts of animal’s bodies (e.g., hair, fur, feathers, nails, beaks, and horns). Due to the large number of reactive functional groups present in its structure, keratin is an excellent candidate for reacting and binding to other materials [13,15].

Keratin has regenerative capacities and can be integrated into biomaterials. It is lightweight, highly available, low cost, ecologically friendly, and insoluble in water, organic solvents, and other common solvents. Additionally, it exhibits good hydrophobic behaviour and has the ability to dampen sound [13].

Wool is a natural fibre commonly found in sheep and other similar animals such as camels, goats, and rabbits, which is obtained through a process known as shearing. This fibre is eco-friendly, sustainable, resistant to bacteria attack, lightweight, recyclable, and has good flame resistance. These qualities make it a valuable raw material [16].

Another fibre with a lot of potential is chiengora or dog wool. Chiengora is spun from the undercoat of a double-coated dog breed, and is extremely soft, waterproof, and warmer than wool, yet less elastic [17]. This fibre is one of the strongest animal fibres, and exhibits high tenacity, good elongation, and good insulating properties [18].

On the other hand, the “green” epoxy resin can be obtained from several natural sources, such as lignin, gallic acid, cardanol, and vegetable oils [19]. Vegetable oils are the most suitable raw materials to synthesize this type of resin. Since they contain unsaturated double bonds, they become a better option for promoting epoxidation reactions [20]. Other “green” resins, such as the biodegradable polyurethane derived from castor oil, have been used to produce composites with cotton fibres [21]. However, not all bio-based resins are biodegradable. The “green” epoxy used in this work is an example of a non-degradable bio-based resin. There is a trend and growing demand in the market for sustainable bio-based materials with an emphasis on their performance, reliability, and strength rather than their biodegradability [22].

Natural fibre-reinforced composites are widely used in the automotive industry. Many car manufacturers have started to use this type of composite in the manufacture of structural parts, because in addition to their strength and durability, they are lightweight, which leads to lower fuel consumption and reduced emissions.

In the end of its life cycle, the component/structure made by these biocomposites can close the carbon loop cycle, if it is disposed by incineration or composting, since the carbon retained during the growth of origin plants is returned to the atmosphere in its disposal.

These materials are also used in construction (e.g., door frames), in electronics, in the manufacture of sports equipment (e.g., tennis rackets, golf clubs, surfboards, bicycles), medical devices, and other industries like aerospace and energy. This diversity of applications highlights the versatility and enormous potential of these materials in reducing environmental impact [1,23,24].

The mechanical behaviour of composite materials is influenced by several factors, including the components selected for reinforcement (the quantity, length, shape, size, composition, orientation, and distribution of the fibres or powders) and the biopolymeric matrix and their interaction, as well as their volume fraction, the mechanical properties of the resin, and the manufacturing techniques used [3]. Because of that, this study was developed with the aim of analysing the influence that different types of natural animal fibres and different fabric production techniques (used as reinforcement) have on the mechanical properties of green composites. From the authors’ knowledge, there are few studies of composites produced with animal fibres, and even fewer with dog hair.

## 2. Materials and Methods

### 2.1. Materials

#### 2.1.1. Fibres

- Serra da Estrela sheep wool

The sheep’s wool yarn used in the production of the composite was obtained commercially from the Burel Factory, without any washing or treatment of the wool. In Table 2 are summarized the properties of sheep’s wool.

- Serra da Estrela dog wool

The dog wool used to produce the green composite was obtained from brushing in the spring, during a Serra da Estrela dog’s change from winter to summer fur. Then, the dog wool was carded and spun to obtain the yarn to produce the reinforcement. The properties of dog wool are indicated in Table 3.

#### 2.1.2. Green Epoxy Resin

The SR GreenPoxy 56 resin, obtained from vegetable oils, was mixed with the SD Surf Clear hardener, both from Sicomin, to create the green composites. The properties of the green resin are summarized in Table 4.

### 2.2. Methods

#### 2.2.1. Fibre Characterization

- X-Ray Diffraction (X-RD)

The X-RD was performed on a Rigaku diffractometer, model DMAX 111/C using CuKα radiation (λ = 1.54 Å) with a voltage of 40 kV and a current of 30 mA. The tests were conducted with a step size of 0.05° and at a rate of 1.2° per minute.

- Tensile tests of the fibre yarns

**Figure 1:**
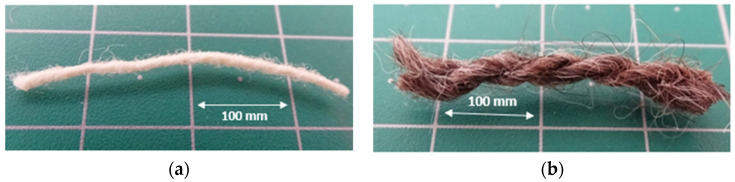
Yarns of the fibres used on the composites—(**a**) Sheep’s wool yarn; (**b**) Dog wool yarn.

The tensile strength of the fibre yarns used was studied, by adapting the ASTM D3822-14 procedure [27]. A Twing-Albert universal mechanical testing machine was used with a 0.5 kN load cell, at a displacement rate of 10 mm/min. The distance between grips was 100 mm and five samples were used to perform the tests. Figure 1 shows the zoomed images of those yarns.

- Scanning Electron Microscope (SEM)

High resolution images of the crocheted fabrics used as reinforcement and the composites produced after the tensile tests were acquired by using a HITACHI model S-3400N scanning electron microscope (SEM).

#### 2.2.2. Production of the Textile Structures

**Figure 2:**
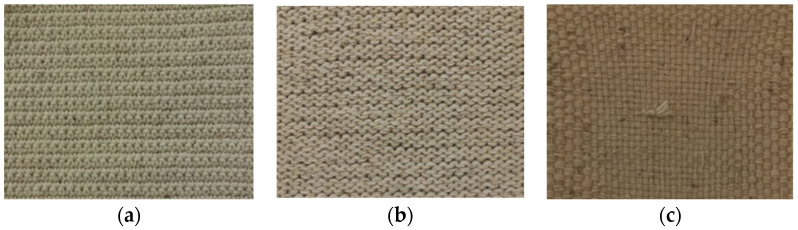
Fabrics produced using sheep’s wool yarns—(**a**) Crocheted fabric; (**b**) Knitted fabric; (**c**) Woven fabric.

**Figure 3:**
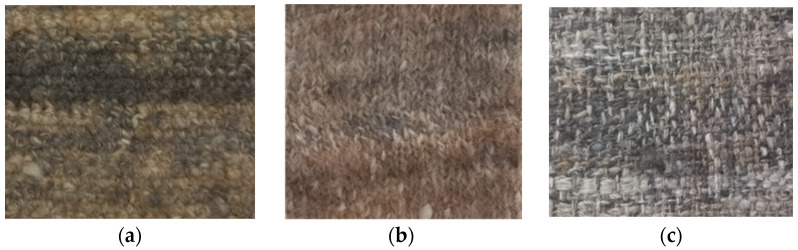
Fabrics produced using dog wool yarns—(**a**) Crocheted fabric; (**b**) Knitted fabric; (**c**) Woven fabric.

In the production of the crocheted fabrics, two different needles were used (needle 3 and needle 5) and the stich used was the single crochet. The knitted fabrics were produced using the knit stitch and only one needle (needle 5). The woven fabrics were created in a taffeta pattern. Figure 2 and Figure 3 show some of the zoomed images of the fabrics produced using both yarns made of dog or sheep wool.

#### 2.2.3. Production of the Composites

**Figure 4:**
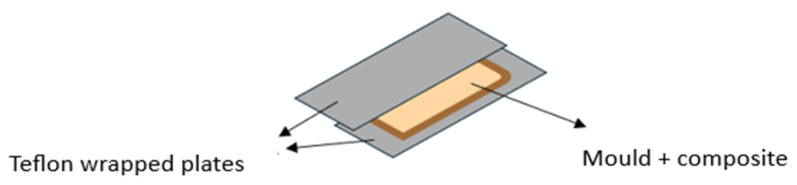
Composite production scheme.

The composites were produced using the hand lay-up moulding process, followed by a confinement in a vacuum bag until its final curing. As can be seen in Figure 4, the first steps in the production of the composites were to wrap the aluminium plates with Teflon and to prepare the mould frame with the following dimensions: 29 cm × 17 cm × 0.1 cm.

After the mould had been properly cut, the resin was mixed with the hardener. Then, the fibres were placed in the mould and the resin was poured over the textile structure until it was completely soaked. Then, the composites were placed under vacuum for 4 h, and curing took place at room temperature after around 24 h. The composites were then post-cured in an oven at 40 °C for 24 h.

The samples produced comply with the following nomenclature: X_V_R_Z, where X refers to the origin of the hair and takes the letters D (dog) and C (sheep); V refers to the type of textile structure and takes the letters C (crochet), K (knitting), and W (weaving); R refers to the number of yarns used and takes the codes 1Y (1 Yarn), 2Y (2 Yarns), and 3Y (3 Yarns); finally, Z refers to the number/size of needle used and takes the codes N3 (needle no. 3) and N5 (needle no. 5).

**Figure 5:**
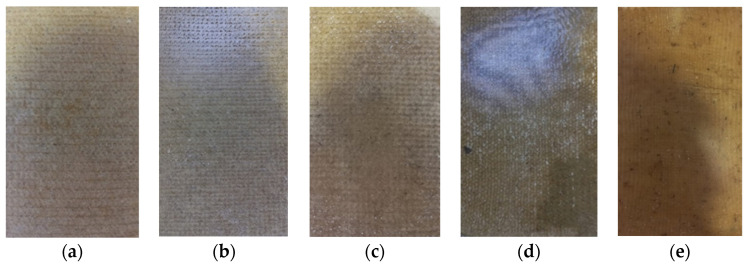
Sheep wool-reinforced composites produced—(**a**) S_C_2Y_N3; (**b**) S_C_2Y_N5; (**c**) S_C_3Y_N5; (**d**) S_K_3Y_N5; (**e**) S_W_3Y.

**Figure 6:**
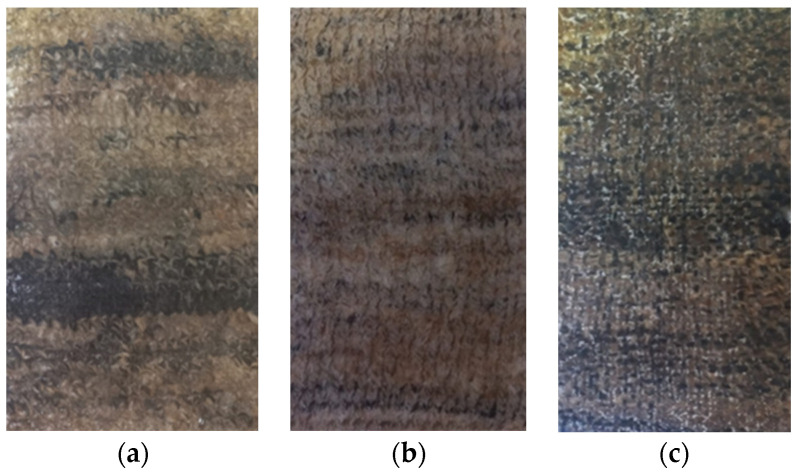
Dog wool-reinforced composites produced—(**a**) D_C_1Y_N5; (**b**) D_K_1Y_N5; (**c**) D_W_1Y.

The different composites produced, from which the specimens needed to carry out the mechanical tests were subsequently cut, are shown in Figure 5 and Figure 6.

The weight fraction of fibres in the composite’s plates can be found in Table 5. To ensure equal mass between the different fibres used, and therefore guarantee a reliable comparison of the composite’s properties, it was necessary to adjust the number of yarns used in the production of the reinforcement. Therefore, three yarns were used in the structure made from sheep wool fibres, while only one yarn was used during the production of the structure made with dog wool fibres.

#### 2.2.4. Mechanical Tests of the Composites

Flexural and tensile tests were carried out to assess the mechanical behaviour of the composites produced. The specimens used in the different tests were cut on a CNC water jet machine. For each type of mechanical test, 5 composite specimens were used.

- Flexural tests

The three-point bending tests were carried out according to the ISO 178:2019 [28], on a Shimadzu universal mechanical test machine, model Autograph AGS-X (Shimadzu Corporation, Kyoto, Japan) with a 10 kN load cell to determine the flexural strength of the specimens. The tests were conducted at a displacement rate of 1.5 mm/min. The span length used in the different tests was carried out according to the standard mentioned above, and the distance between the supports used during the flexural tests is described in Table 6.

- Tensile tests

**Figure 7:**
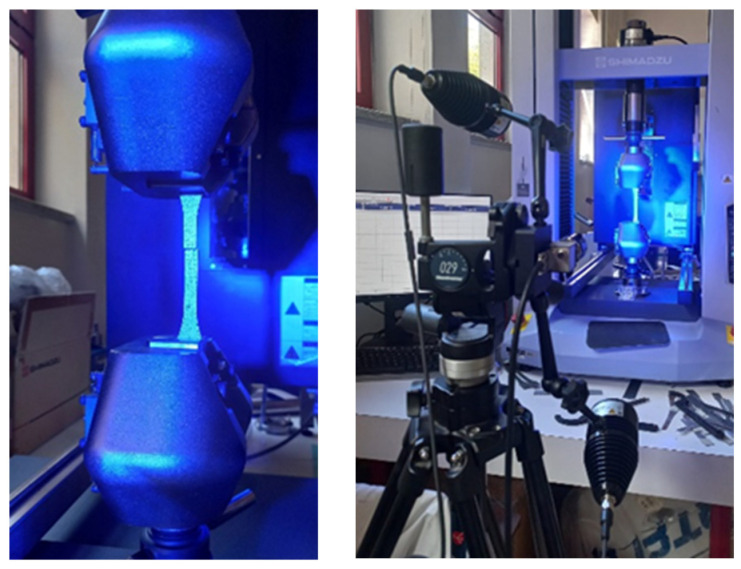
Tensile test and Digital Image Correlation setup.

The tensile tests were carried out in accordance with the ISO 527-1 [29] and ISO 527-4 [30] using a Shimadzu universal mechanical test machine (Shimadzu Corporation, Kyoto, Japan) with a 50 kN load cell, at a displacement rate of 2 mm/min. The distance between grips was 100 mm. Strain measurements of tensile tests were obtained using Digital Image Correlation (GOM system), as the test setup presented in Figure 7.

## 3. Results

### 3.1. Fibre Characterization

#### 3.1.1. X-Ray Diffraction (X-RD)

**Figure 8:**
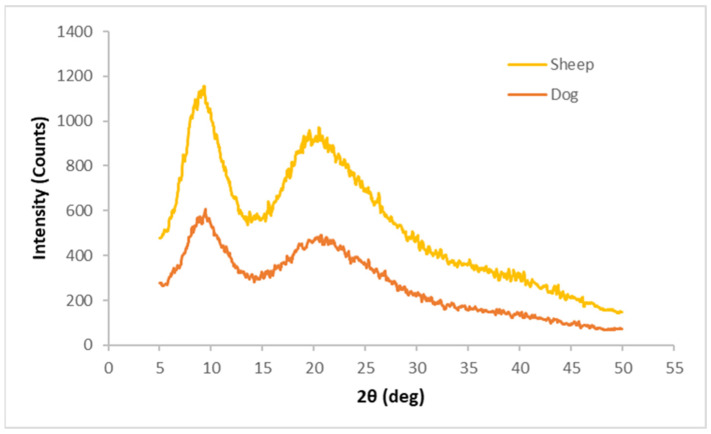
X-RD patterns for Serra da Estrela dog and sheep wool fibres.

The X-RD patterns obtained for both fibres are shown in Figure 8.

#### 3.1.2. Tensile Tests of the Fibres Yarns

**Figure 9:**
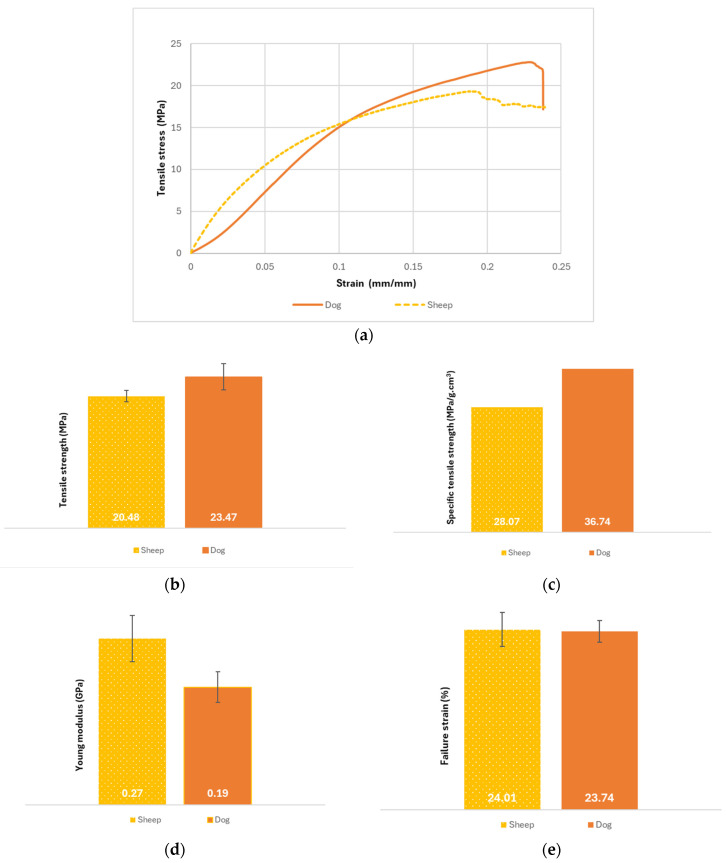

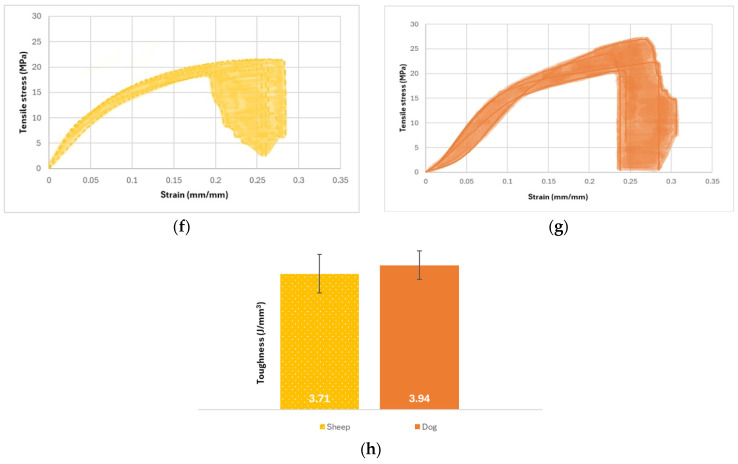
Results of the tensile tests of the yarns—(**a**) Average tensile stress/strain graph; (**b**) Tensile strength results (MPa); (**c**) Specific tensile strength results (MPa/g·cm^3^); (**d**) Tensile Young’s modulus (GPa); (**e**) Failure strain results (%); (**f**) Evolute of tensile stress/strain curves for sheep wool yarn; (**g**) Evolute of tensile stress/strain curves for dog wool yarn; (**h**) Toughness or deformation energy accumulated until the first failure (J/mm^3^).

The graphs of the average tensile stress/strain curves, the tensile strength results, the specific tensile strength results, the tensile Young’s modulus values, the failure strain values, the evolute of tensile stress/strain curves, and the toughness of the yarns used to produce the reinforcements are shown in Figure 9.

#### 3.1.3. Scanning Electron Microscope (SEM) of the Crocheted Fabrics

**Figure 10:**
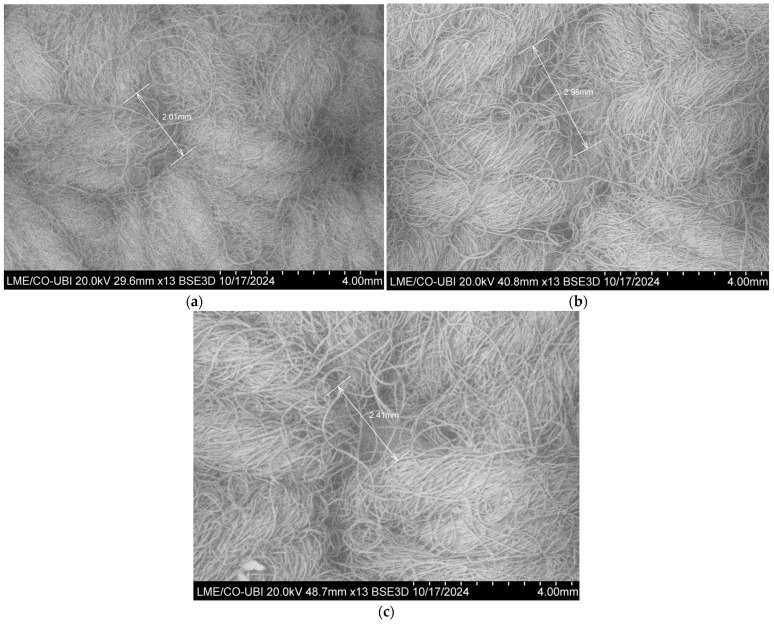
SEM images of the crocheted fabrics used as reinforcement of the composites—(**a**) 2Y_N3; (**b**) 2Y_N5; (**c**) 3Y_N5.

The high-resolution images obtained of the crocheted fabrics used as reinforcement are shown in Figure 10. Also, in this figure are indicated the diameters of the stiches produced by the different needles.

### 3.2. Mechanical Tests of the Composites

#### 3.2.1. Flexural Tests

**Figure 11:**
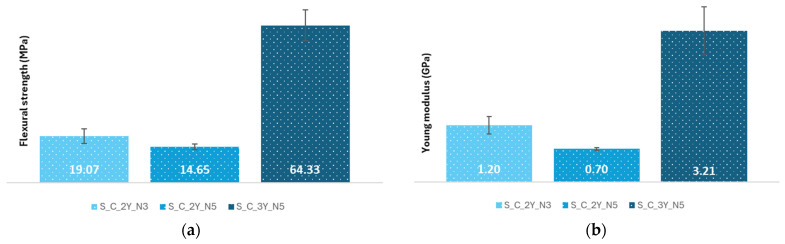
Results of the flexural tests of the composites reinforced with crocheted fabrics made from sheep wool—(**a**) Flexural strength results (MPa); (**b**) Young’s modulus results (GPa).

The flexural strength results, and the flexural Young’s modulus values for the composites reinforced with fabrics crocheted from sheep’s wool are shown in Figure 11.

**Figure 12:**
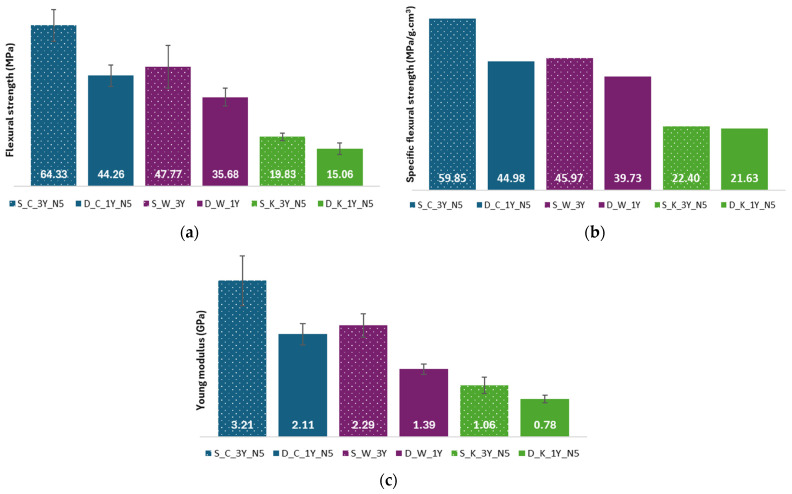
Results of the flexural tests of the composites reinforced with different types of textile structures—(**a**) Flexural strength results (MPa); (**b**) Specific flexural strength results (MPa/g·cm^3^); (**c**) Young’s modulus results (GPa).

The flexural strength results, the specific tensile strength results, and the flexural Young’s modulus values for the composites reinforced with different types of textile structures are shown in Figure 12.

#### 3.2.2. Tensile Tests

**Figure 13:**
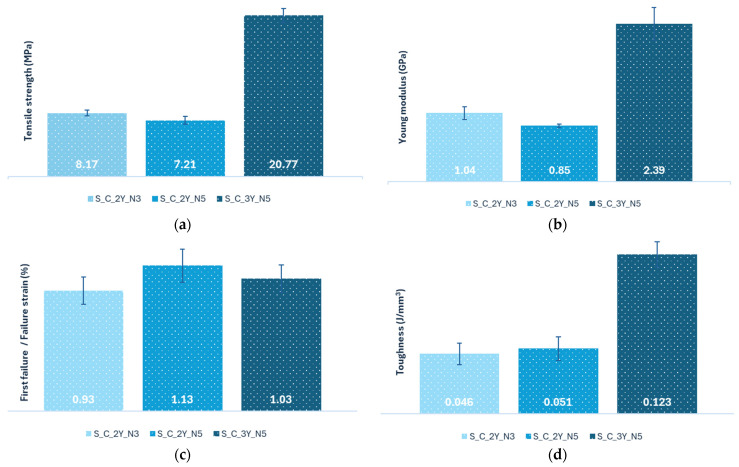
Results of the tensile tests of the composites reinforced with crocheted fabrics made from sheep’s wool—(**a**) Tensile strength results (MPa); (**b**) Young’s modulus results (GPa); (**c**) First failure/Failure strain results (%); (**d**) Toughness results (J/mm^3^).

The results for tensile strength, the values of tensile Young’s modulus, the first failure/failure strain values, and the toughness of the composites reinforced with fabrics crocheted from sheep’s wool are shown in Figure 13.

**Figure 14:**
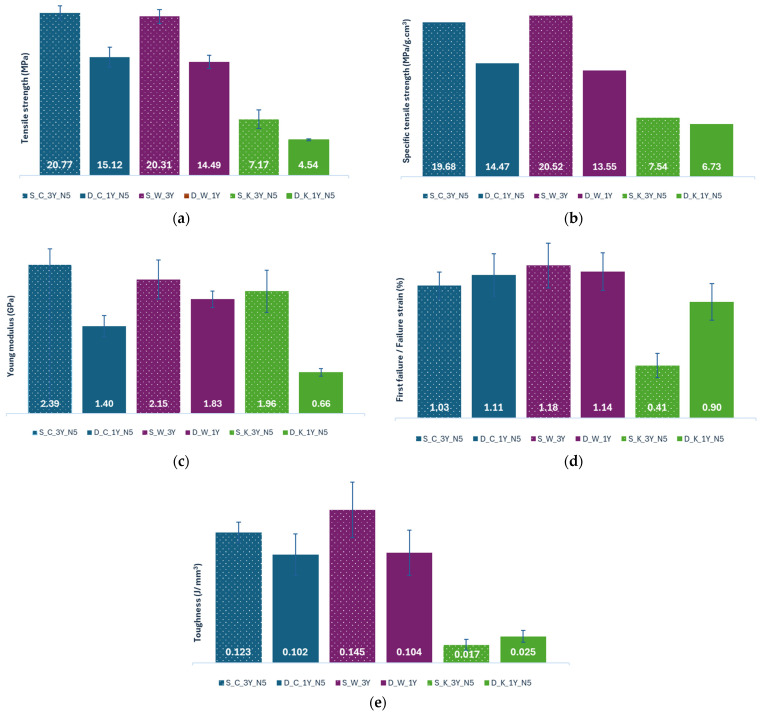
Results of the tensile tests of the composites reinforced with crocheted fabrics made from sheep’s wool—(**a**) Tensile strength results (MPa); (**b**) Specific tensile strength results (MPa/g·cm^3^); (**c**) Young’s modulus results (GPa); (**d**) First failure/Failure strain results (%); (**e**) Toughness results (J/mm^3^).

The tensile strength results, the results for specific tensile strength, the values of tensile Young’s modulus, the first failure/failure strain results, and the toughness of the composites reinforced with different types of textile structures are illustrated in Figure 14.

The rule of mixtures enables us to calculate the limit values for the global modulus of elasticity of the composite. Considering the isostress and isostrain conditions of a composite, where the load is parallel or transverse to the fibres, respectively, the limit values for the elastic modulus of the composite are obtained by Equations (1) and (2), respectively.

(1) $$E_{C,T}=\frac{E_{m}\cdot E_{f}}{V_{m}\cdot E_{f}+V_{f}\cdot E_{m}}\;$$

where $E_{C,T}$ is the elastic modulus, obtained considering the isostress condition, $E_{m}$ and $E_{f}$ are the elastic modulus of the matrix and fibre, respectively, in N/mm^2^, and $V_{m}$ and $V_{f}$ are the volume fractions of the matrix and fibre, respectively.

(2) $$E_{C,L}=E_{m}\cdot V_{m}+E_{f}\cdot V_{f}$$

where $E_{C,L}$ is the elastic modulus, obtained considering the isostrain condition, $E_{m}$ and $E_{f}$ are the elastic modulus of the matrix and fibre, respectively, in N/mm^2^, and $V_{m}$ and $V_{f}$ are the volume fractions of the matrix and fibre, respectively. The values of composite volume fractions of matrix and fibres, and stiffness of matrix and fibres used in the rule of mixtures, obtained from measuring the reinforcement and the final laminate weights, are presented in Table 7 and Table 8 show the elastic modulus obtained considering the isostress and the isostrain conditions, as well as the experimentally obtained values.

### 3.3. Scanning Electron Microscope (SEM) of the Composites

**Figure 15:**
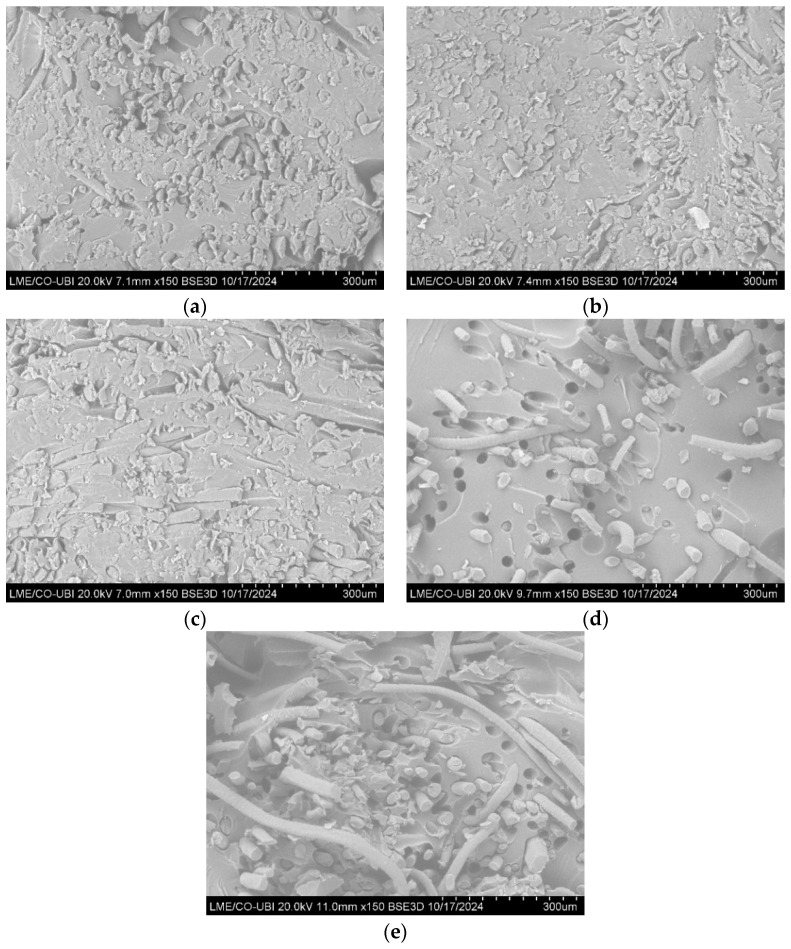
SEM images of the composites reinforced with sheep wool—(**a**) C_2Y_N3; (**b**) C_2Y_N5; (**c**) C_3Y_N5; (**d**) K_3Y_N5; (**e**) W_3Y.

**Figure 16:**
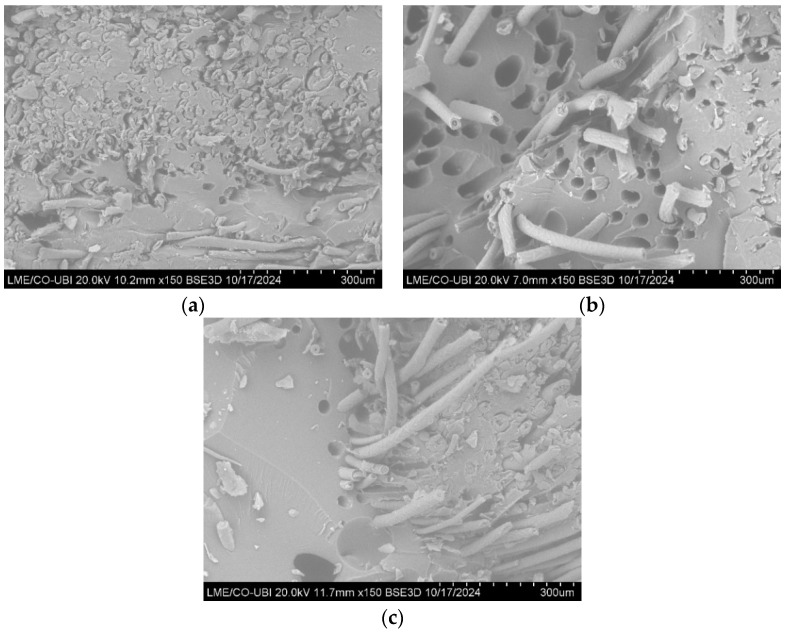
SEM images of the composites reinforced with dog wool—(**a**) C_1Y_N5; (**b**) K_1Y_N5; (**c**) W_1Y.

The high-resolution images of the composites produced are shown in Figure 15 and Figure 16.

## 4. Discussion

### 4.1. Fibre Characterization

#### 4.1.1. X-Ray Diffraction (X-RD)

By analysing the X-RD patterns obtained for the sheep’s wool and dog wool (Figure 8), one can observe, in both fibres, two strong diffraction peaks at the 2θ angles at around 9° and around 20°, corresponding to the α-helix and β-sheet structures of keratin, respectively. One can also see that the diffraction peak at the 2θ angle around 20° has a broader shape, which might be caused by the overlap of the β-sheet structures [21,31,32].

When one compares both fibres, one can see that the dog wool fibre has a lower diffraction intensity in both peaks, which suggests that dog wool is less crystalline than sheep’s wool [32]. Sheep wool fibre has a degree of crystallinity of 58.82% and dog wool fibre has 56.60%.

#### 4.1.2. Tensile Tests of the Yarns

From the graph of Figure 9a, one can see that both yarns have similar maximum tensile stress values, with overlapping evolutes (see Figure 9f,g). Also, it was observed, during the tensile tests, that after reaching the peak tensile stress, the dog wool breaks immediately (sudden drop in tensile stress after reaching the maximum value). Meanwhile, the sheep’s wool yarn does not break after reaching the maximum tension. In fact, one cannot affirm, from analysing the graph, that the yarn breaks completely. What one sees is a gradual decrease in the tensile stress value, which suggests that the fibres within the yarn are breaking one by one, as they slide between them.

Analysing the results obtained for the tensile strength of the yarns shown in Figure 9b, it is possible to observe that the yarn made from dog wool has a higher average value than the yarn produced from sheep’s wool (23.47 MPa and 20.48 MPa, respectively).

Regarding the specific tensile strength (Figure 9c), dog wool yarn has a value of 36.74 MPa/g·cm^3^ and sheep’s wool yarn has a value of 28.07 MPa/g·cm^3^. According to the data shown in Figure 9b,c, one can see that dog wool yarn is more resistant than sheep’s wool yarn.

According to Figure 9d, the sheep’s wool yarn has a higher Young’s modulus (0.27 GPa) than dog wool yarn (0.19 GPa). These results were expected, although the values obtained are significantly lower than anticipated (Table 2 and Table 3). Based on these results, one can conclude that sheep’s wool is stiffer than dog wool.

From Figure 9e, one can observe that the sheep’s wool yarn has a higher value of the strain at failure (24.01%) than dog wool yarn (23.74%). Based on these values, one can say that sheep wool fibres are able to deform more and are able to sustain a greater load before breaking than dog wool fibres.

From Figure 9h, one can observe that the deformation energy accumulated until the first failure, or the first drop in stress, was higher for the dog wool yarn with a value of 3.94 J/mm^3^. These results suggest that dog wool fibres are more tenacious and can absorb more energy before fracturing than sheep wool fibres.

### 4.2. Mechanical Tests of the Composites

Figure 11 and Figure 13 demonstrate the influence that the number of yarns and the diameter of the needle used have on the mechanical properties of the composites, and Figure 12 and Figure 14 demonstrate the impact that the type of fibre and the type of fabric structure used as reinforcement have in the tensile and flexural properties of the composites.

#### 4.2.1. Flexural Tests

According to Figure 11a, the S_C_3Y_N5 composite has the highest flexural strength value (64.33 MPa) and the S_C_2Y_N5, with a value of 14.65 MPa, has the lowest one. Comparing the S_C_2Y_N5 and S_C_3Y_N5 composites, it is possible to observe that increasing the number of yarns used in the production of the fabrics leads to a significant increase in the flexural strength, since the S_C_3Y_N5 composite has a significantly higher average value than the S_C_2Y_N5 composite (64.33 MPa and 14.65 MPa, respectively).

**Figure 17:**
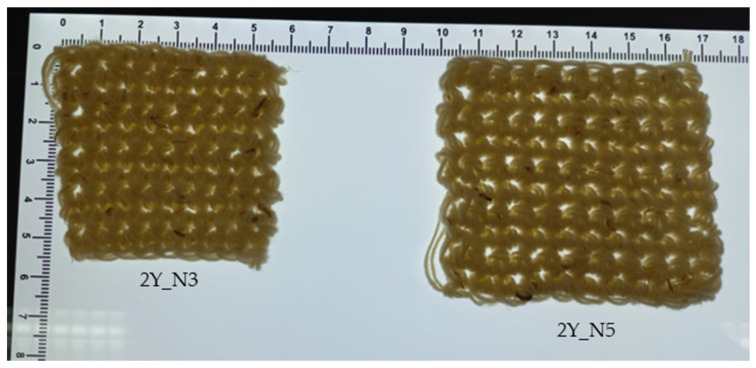
Crocheted fabrics produced using the yarns made of sheep’s wool and different needles.

Regarding the influence of the needle diameter on this mechanical property (Figure 11a), one can see that the S_C_2Y_N3 composite exhibits a higher flexural strength than that of the S_C_2Y_N5 composite (19.07 MPa and 14.65 MPa, respectively), which would be expected, since needle 3 has a smaller diameter than needle 5. A needle with a smaller diameter makes a more closed microstructure of the reinforcement, and tighter stitches, so the meshes produced are less flexible and less elastic, which increases the overall strength and stiffness of the composite (Figure 10a,b and Figure 17).

Figure 17 shows two crocheted fabrics made from sheep’s wool with the exact same number of stitches present (10 × 10). From this figure, one can see that the stitches produced by needle 3 are tighter, since the structure obtained is more closed and smaller in size. By analysing Figure 10a,b, one can again confirm that claim, since needle 3 produced stitches with a diameter of 2.01 mm and needle 5 produced stitches with a diameter of 2.98 mm.

As for the Young’s modulus (Figure 11b), it can be noticed that the composite with the highest value is the S_C_3Y_N5 composite (3.21 GPa), and the composite S_C_2Y_N5 has the lowest value (0.7 GPa). From this figure, one can conclude that increasing the number of yarns used leads to a significant increase in the stiffness of the composite material. Additionally, reducing the needle diameter, while keeping the number of the yarns used the same, positively affects the toughness of the composites.

The highest average value for the flexural strength and Young’s modulus obtained for the composite S_C_3Y_N5 can be explained due to the use of a larger number of yarns, even though needle 5 was used. This suggests that the higher number of yarns used in the reinforcement compensates for the flexibility caused by the use of needles having a bigger diameter. The S_C_2Y_N5 composite has the lowest value due the fact that needle 5 and a smaller number of yarns were used to produce the reinforcement (Figure 11a,b).

From Figure 12a, one can see that the composite S_C_3Y_N5 exhibits the highest flexural strength average value (64.33 MPa) and the D_K_1Y_N5 composite has the lowest value (15.06 MPa). Analyzing the results of the flexural strength in this figure, one can see that the composites reinforced with sheep’s wool have higher flexural strength average values than those reinforced with dog wool (comparison between the same fabric production technique), which was expected due the higher number of yarns used. And comparing the different fabric production techniques, the composites reinforced with crocheted structures have the highest flexural strength values, while the ones reinforced with knitted fabrics have the lowest. Weaving, due to the orientation of the yarns, is a structure with more dimensional stability than crochet and knitting [33], so it was expected that of all the composites, those reinforced with weaving fabrics would be the ones with the highest bending strength, but this is not the case. This can be explained due to the high fibre fraction of the composites reinforced with crocheted meshes (Table 5). The low flexural strength of the composites reinforced with knitted fabrics can be justified, considering the high flexibility of these fabrics and the low fibre fraction of the composites reinforced with these fabrics (Table 5).

Comparing Figure 12a,b, one can see that there are no significant differences between them. The S_C_3Y_N5 composite has the highest specific flexural strength (59.85 MPa/g·cm^3^) and the D_K_1Y_N5 composite has the lowest average value (21.63 MPa/g·cm^3^), although it is very similar to the value for the S_K_3Y_N5 composite (22.40 MPa/g·cm^3^).

Comparing the types of fibres used to produce the reinforcement, composites reinforced with sheep wool fibres have higher specific flexural strength values than those reinforced with dog wool fibres. As for the influence of the type of fabric used as reinforcement material on this mechanical property, one can see that the composites reinforced with crocheted fabrics have the highest specific flexural strength values, followed by those reinforced with fabrics made from weaving, and the composites reinforced with knitted fabrics have the lowest specific flexural strength values (Figure 12b).

According to Figure 12c, the S_C_3Y_N5 composite has the highest Young’s modulus (3.21 GPa) and the D_K_1Y_N5 composite has the lowest (0.78 GPa). Also, one can see that the composites reinforced with sheep’s wool have higher Young’s modulus values than those reinforced with dog wool (comparison made between composites reinforced with the same type of textile structure). This can be explained due to the higher number of yarns used in the production of the fabrics reinforced with sheep’s wool and to the fact that sheep’s wool is stiffer than dog wool (Figure 9d and Table 2 and Table 3). Comparing the different techniques used in the production of the reinforcement, composites reinforced with crocheted fabrics have the highest Young’s modulus values and those reinforced with knitted structures have the lowest values. These results can be attributed to the higher fibre content present in the composites reinforced with the crocheted fabrics and to the high flexibility of the knitted meshes, combined with the lower fibre percentage in the composites reinforced with the knitted meshes.

The highest value for the tensile strength, specific tensile strength, and Young’s modulus obtained for the S_C_3Y_N5 composite can be explained by the use of a greater number of yarns and to the higher fibre content (Table 5). The composite D_K_1Y_N5 has the lowest values due to the high flexibility of the knitted fabrics and to the lower number of yarns used (Figure 12a–c).

Comparing the results obtained for the flexural strength and Young’s modulus (Figure 12a,c) for the composites reinforced with dog wool with the results obtained by Gomes et al. [19] for the composites made with green epoxy matrix reinforced with dog wool without any treatment, one can see that the transformation of the fibres in yarns, and subsequently in fabrics, improved both mechanical properties, with the exception of the composite D_K_1Y_N5. This improvement was expected, because the random distribution of the fibres in the composites produced by Gomes et al. does not allow for the load applied to the composites to be distributed in the most efficient way, which results in a reduction in the mechanical properties of the composite materials.

#### 4.2.2. Tensile Tests

Analyzing Figure 13a,b, one can see that the composites maintain the same behaviour as in the flexural tests. According to Figure 13a, the composite S_C_3Y_N5 exhibits the highest tensile strength value (20.77 MPa), and the S_C_2Y_N5 composite exhibits the lowest one, with a value of 7.21 MPa. Analyzing the composites S_C_2Y_N5 and S_C_3Y_N5, it is possible to observe that the tensile strength increases significantly with the increase in number of yarns used to produce the reinforcement. As to the influence of the needle diameter on this mechanical property, one can see that the S_C_2Y_N3 composite exhibits a higher tensile strength compared to the S_C_2Y_N5 composite (8.17 MPa and 7.21 MPa, respectively). This outcome was expected, given that needle 3 has a smaller diameter than needle 5, and a needle with a smaller diameter creates tighter stiches, so the meshes produced are less flexible and less elastic (Figure 10a,b and Figure 17), which consequently increases the resistance of the composite. However, this composite presented a more fragile behaviour, being less tough (0.046 J/mm^3^ and 0.051 J/mm^3^, respectively) (Figure 13d).

Regarding the Young’s modulus results (Figure 13b), one can see that the composite with the highest value is the S_C_3Y_N5 composite, with a value of 2.39 GPa, and the composite S_C_2Y_N5 has the lowest value (0.85 GPa). This figure suggests that increasing the number of yarns used in the reinforcement and decreasing the diameter of the needle used, while maintaining the number of yarns used constant, increases the stiffness of the composite material.

The S_C_2Y_N5 composite has the lowest tensile strength and Young’s modulus values (Figure 13a,b) because, in addition to the lowest number of yarns used, the needle used was needle 5 (the one with the largest diameter), so the meshes produced are more flexible, and therefore the composite produced is less resistant and less stiff. On the other hand, the S_C_3Y_N5 composite has the highest tensile modulus and tensile strength of the three composites because, during the production of the reinforcement, a greater number of yarns was used.

Analyzing Figure 13c, one can observe that the composite S_C_2Y_N5 has the highest strain at first failure/at break, with a value of 1.13%, while the S_C_2Y_N3 has the lowest value (0.93%). The fact that the S_C_2Y_N5 composite is the one with the highest deformation value at first failure/at break can be explained due to the fact that it uses less yarns in the reinforcement and uses a needle with a larger diameter, which makes the fabric less resistant and more flexible. In addition, this composite has the lowest Young’s modulus of the three composites analyzed (Figure 13b); in other words, it is the least stiff of the three, which might indicate that it has some ductility and therefore is able to withstand a larger amount of deformation before reaching its breaking point.

Comparing the composites S_C_3Y_N5 and S_C_2Y_N5, one can see that the first one has a lower first failure/break strain value (1.03% and 1.13%, respectively). These values can be explained by the fact that the S_C_3Y_N5 composite was produced having a higher number of yarns, which increases the composite’s stiffness and strength, but reduces the strain at failure. A higher stiffness might indicate that the material is less ductile, and therefore it might even crack abruptly, which translates into a lower percentage of deformation. Analyzing the composites S_C_2Y_N3 and S_C_2Y_N5, one can observe that the S_C_2Y_N5 composite has a higher value for the strain at break/first failure (1.13%) in comparison with the composite S_C_2Y_N3 (0.93%). The higher value of the S_C_2Y_N5 composite can be explained by the use of a needle with a larger diameter in the production of the reinforcement, which produces a more flexible fabric that leads to the production of less stiff composites. Hence, the material will deform more before breaking (Figure 13c).

According to Figure 13d, one can observe that the composite S_C_3Y_N5 exhibits the highest toughness, with a value of 0.0123 J/mm^3^, and the composite S_C_2Y_N3 exhibits the lowest one (0.046 J/mm^3^). Comparing the composites S_C_3Y_N5 and S_C_2Y_N5, it is possible to see that the first one has a higher toughness value (0.123 J/mm^3^ and 0.051 J/mm^3^, respectively); in other words, the composite S_C_3Y_N5 can absorb more energy before rupturing than the S_C_2Y_N5 composite. These values can be explained by the use of a greater number of yarns in the production of the reinforcement.

Regarding the composites S_C_2Y_N3 and S_C_2Y_N5, one can verify that the first one has a lower toughness (0.046 J/mm^3^ and 0.051 J/mm^3^, respectively). Given the fact that between these two composites, the S_C_2Y_N3 is the one with the higher tensile strength and rigidity, these results do not align with what was expected. The production of the S_C_2Y_N3 composite used the needle with the smaller diameter; therefore, the meshes produced are less flexible and less elastic, and the stitches made by this needle are tighter than those produced by needle 5, so it was anticipated that the composite S_C_2Y_N3 would be capable of absorbing a larger amount of energy up to its breaking point than the composite S_C_2Y_N5 (Figure 13d).

Analyzing the tensile strength results in Figure 14a, one can observe that the S_C_3Y_N5 composite has the highest tensile strength value of 20.77 MPa, while the composite D_K_1Y_N5 has the lowest value (4.54 MPa). Also, the S_W_3Y composite has a value (20.31 MPa) very similar to the S_C_3Y_N5 composite. The higher tensile strength value of the S_C_3Y_N5 composite can be explained by the number of yarns used and by the higher fibre content present (Table 5), while the lower value of the D_K_1Y_N5 composite can be explained by the high flexibility of the reinforcement and by the lower number of yarns used.

From the analysis of Figure 14a, it is evident that the composites reinforced with sheep’s wool are more resistant than the ones reinforced with dog wool, which was expected due to the higher number of yarns used. Comparing the different reinforcement manufacturing techniques, one can see that the composites reinforced with crocheted structures have the highest tensile strength values, followed by those reinforced with woven fabrics, while the ones reinforced with knitted meshes have the lowest values. Knitted and crochet fabrics are more flexible and elastic than woven fabrics [16,33], so theoretically, composites reinforced with these fabrics should have lower strength and stiffness values than the composites reinforced with the fabrics made by weaving, but this is not the case. The highest strength of the composites reinforced with crocheted fabrics can be explained by the high quantity of fibres present (Table 5), even though these structures have more flexibility and less dimensional stability than woven fabrics. On the other hand, the low strength of the composites reinforced with the knitted fabrics can be explained by the high elasticity of the reinforcement (more elastic than crocheted fabric [33]) and by the low amount of fibres present in the composite materials (Table 5).

According to Figure 14b, one can see that the composite with the highest specific tensile strength value is S_W_3Y, with a value of 20.52 MPa/g·cm^3^, followed by S_C_3Y_N5 with a value of 19.68 MPa/g·cm^3^. And the composite with the lowest specific tensile strength value is D_K_1Y_N5 (6.73 MPa/g·cm^3^), with a value very similar to the S_K_3Y_N5 composite (7.54 MPa/g·cm^3^). The fact that the composite S_W_3Y has the highest specific tensile strength can be explained by the higher number of yarns used and the more dimensional stable fabrics used as reinforcement. In contrast, the lower value of the D_K_1Y_N5 composite can be attributed to the combination of the high flexibility of the reinforcement, the low fibre content (Table 5), and the lower number of yarns used in the manufacture of the reinforcement.

When comparing the fibres used in the reinforcement, the composites reinforced with sheep wool fibres exhibit a higher specific tensile strength value than those reinforced with dog wool fibres. These results were expected, considering the higher number of yarns used in the composites reinforced with sheep’s wool. Regarding the impact of the textile structure used as reinforcement on the composite mechanical properties (Figure 14b), one can observe that composites reinforced with crocheted and woven fabrics have the highest specific tensile strength values and those reinforced with knitting fabrics have the lowest values. These results can be explained by the architecture of the reinforcement and by the amount of fibres present.

Analyzing the Young’s modulus results from Figure 14c, one can notice that the S_C_3Y_N5 composite has the highest value of 2.39 GPa, and the D_K_1Y_N5 has the lowest (0.66 GPa). The fact that the S_C_3Y_N5 composite has the highest value can be explained by the higher number of yarns used, the higher stiffness of the fibres (Figure 9d and Table 2 and Table 3), and the high fibre content present (Table 5). The lowest stiffness of the D_K_1Y_N5 composite can be attributed to the lower stiffness of the fibres, the greater elasticity of the reinforcement, and the lower number of yarns used during its production. Comparing the two types of fibres used in the reinforcement, one can observe that the composites reinforced with sheep wool fibres have a higher Young’s modulus than those reinforced with dog wool fibres. These higher values could be due to the fact that the composites produced with sheep’s wool are reinforced with a bigger number of yarns and to the fact that sheep’s wool is stiffer than dog wool.

Regarding the influence of the architecture of the reinforcement on the modulus of elasticity (Figure 14c), one can see that in the composites where dog wool was used, the ones reinforced with woven fabrics have the highest Young’s modulus. This can be explained by the fact that weaving produces fabrics’ dimensional stability by the considerable amount of fibres present (Table 5). In the composites where sheep’s wool was used, those reinforced with crocheted fabrics are the stiffest composites. This result can be attributed to the high fibre fractions present in the composites reinforced with crocheted meshes, which compensates for the higher stability of the woven fabrics. For both types of fibres, the composites reinforced with the knitted fabrics are less rigid. This can be attributed to the fact that knitting produces more elastic fabrics, which consequently reduces the stiffness of the composite material, and due to the lower fibre content present in these composites.

Comparing the results obtained for the tensile strength and Young’s modulus (Figure 14a,c) for the composites reinforced with dog wool with the results obtained by Gomes et al. [19] for the composites made with green epoxy matrix reinforced with dog wool without any treatment, it is possible to observe that the transformation of the fibres in yarns, and subsequently in fabrics, improved both mechanical properties, with the exception of the composite D_K_1Y_N5. This improvement was expected, because the random distribution of the fibres in the composites produced by Gomes et al. does not allow for the load applied to the composites to be distributed in the most efficient way, which results in a reduction in the mechanical properties of the composite materials.

Analysing Figure 14d, one can observe that the composites S_C_3Y_N5, D_C_1Y_N5, S_W_3Y, and D_W_1Y have very close values and S_W_3Y has the highest strain at first failure/break value (1.18%), while the composite S_K_3Y_N5 has the lowest value (0.41%). The S_K_3Y_N5 composite has the lowest quantity of fibres (Table 5) of all the composites and is also reinforced with a knitted fabric, the fabric with the highest flexibility of all the fabrics studied. For those reasons, this composite will have a low strength and stiffness, and the composite will exhibit an elastic behaviour, with the applied load being mostly supported by the polymeric matrix. Regarding the composite O_W_3Y, due to the geometry of the reinforcement fabric, this composite has a high strength and stiffness, so it should have a lower deformation value. The high value for the deformation at first failure/break obtained for this composite could be explained by the low fraction of fibres present; in other words, the load applied is mostly sustained by the polymeric matrix, which is less strong and stiff than the reinforcement.

Comparing the fibres used in the reinforcement, composites reinforced with sheep’s wool exhibit higher strain at first failure/break values if the reinforcement was made by weaving, but if the reinforcement was made by crochet or knitting, the composites reinforced with dog wool exhibit the highest values. Due to the superior strength and stiffness of the composites reinforced with sheep’s wool, they should display lower deformation values at first failure/break than those reinforced with dog wool, no matter which type of reinforcement is used. This applies except for the composites reinforced with woven fabrics, where the composites reinforced with dog wool fibres have higher strain at first failure/break values than those reinforced with sheep wool fibres (Figure 14d).

Regarding the impact of the type of textile structure used as reinforcement on the deformation at first failure/break, one can see that the composites reinforced with woven fabrics have the highest deformation values, followed by the composites reinforced with crocheted fabrics, and the composites reinforced with knitted structures have the lowest values. The results obtained are not in accordance with the expected results, since due to the high dimensional stability of the woven fabrics and to the high strength and stiffness of the composites reinforced with these structures, the percentage of deformation at first failure/break should be lower. It was expected, in fact, to show the lowest value of all the composites analysed. In composites reinforced with woven structures, the loads applied are distributed in two directions (along the direction of the weft and warp yarns) [34], while in composites reinforced with meshes, the loads applied can be distributed in several directions, which leads to less stiff behaviour.

According to Figure 14e, one can verify that the composite S_W_3Y has the highest toughness value (0.145 J/mm^3^), and S_K_3Y_N5 has the lowest value (0.017 J/mm^3^). The highest value of the S_W_3Y composite can be explained by the higher number of yarns used, the higher rigidity of the fibres, and to the dimensional stability of the reinforcement, while the lowest value of the composite S_K_N5 can be explained by the high flexibility of the reinforcement, and by the low quantity of fibres present (Table 5). From the analysis of this figure, it is possible to verify that the composites reinforced with sheep’s wool have higher toughness values than those reinforced with dog wool, except when the reinforcement was made by knitting. These values were expected, since the composites reinforced with sheep wool fibres have higher tensile strength and stiffness than the ones reinforced with dog wool fibres, and therefore are able to absorb more energy until breaking (Figure 14a–c). The fact that the composite S_K_3Y_N5 is less tenacious than the D_K_1Y_N5 composite may be due to the lower fibre content present in the first one (Table 5).

Comparing the different reinforcement manufacturing techniques, the composites reinforced with woven fabrics are the ones that absorb more energy before breaking, followed by the composites reinforced with crocheted fabrics. The composites reinforced with knitted meshes are the ones with the lowest toughness values. The highest values of the composites reinforced with the woven fabrics can be explained by the great stability of these fabrics, while the lowest values of the composites reinforced with the knitted fabrics can be explained by the high flexibility of the meshes and to the low quantity of the fibres present in those composites (Figure 14e).

It is important to emphasize that the strain values presented were obtained in different contexts: deformation at first failure and deformation until complete rupture of the composites. Also, it is important to note that in the analysis of the results obtained (Figure 13c and Figure 14d), there was no distinction between the specimens that broke completely and those that at the end of the tensile test still had intact fibres.

Analyzing the results from Table 8, one can verify that the Young’s modulus results obtained experimentally for the different composites are in between the limits predicted by the theoretical models of the rule of mixtures, considering isostress and isostrain conditions, except the composite D_K_1Y_N5, where the elastic modulus obtained experimentally is equal to the modulus considering the isostress condition. Considering these results, one can conclude that the composites have a stiffness intermediately between the stiffness of the fibres and the matrix. Since the fibres’ stiffness is very low, compared to the resin matrix stiffness, the resin will not be reinforced in terms of stiffness.

### 4.3. SEM of the Composites

By analyzing Figure 15 and Figure 16, it is possible to identify some of the failure mechanisms that occurred during the tensile tests.

Comparing the different types of structures used as reinforcement, one can see that in the composites reinforced with crocheted fabrics (Figure 15a–c and Figure 16a), there are less holes present in the resin, which indicates that there was a low occurrence of fibre pull-out. One can also see that the main failure mechanism in this type of composite is the fibre fracture. In contrast, in the composites reinforced with woven and knitted fabrics (Figure 15d,e and Figure 16b,c), it is possible to see a considerable number of holes in the matrix (higher number of holes present in the composites reinforced with knitted fabrics), which indicates the occurrence of fibre pull-out.

These results suggest that the interfacial interaction between the fibres and the matrix of composites reinforced with crocheted fabrics is greater than the interaction between composites reinforced with woven and knitted fabrics. The higher the interaction between the two components, the greater the mechanical performance of the composite material, a claim that was demonstrated in the mechanical tests carried out.

## 5. Conclusions

In this work, eight green composites reinforced with sheep and dog wool fibres, both from Serra da Estrela, were produced in order to study their flexural and tensile behaviours.

The main results obtained can be divided into two parts. The first part, where only the mechanical test results of the composites reinforced with crocheted fabrics made with sheep’s wool were studied, concerns the influence that the diameter of the needle and the number of yarns used in the production of the reinforcement have on the tensile and flexural properties of the composites produced. The second part focuses on the influence that the type of fibre and the fabric architecture have on these two mechanical properties.

1. Influence of the needle diameter and number of yarns used
  - Increasing the number of yarns used increases the flexural strength (by 339.11%), the tensile strength (188.07%), and stiffness of the composites;
  - Reducing the diameter of the needle used in the reinforcement increases the flexural strength (by 30.17%), the tensile strength (13.31%), and stiffness of the composites;
  - Increasing the number of yarns used in the reinforcement increases the composite’s stiffness, which might reduce the ductility of the composite material, and therefore allow the material to endure a lower deformation before breaking (decrease of 9.71%);
  - The use of a needle with a larger diameter produces more flexible fabrics, which decreases the stiffness of the composites and allows the material to deform more before breaking (increase of 21.51%);
  - Increasing the number of yarns used in the reinforcement increases significantly the toughness of the composites by 141.18% and the reduction in the diameter of the needle used decreases the tenacity by 10.87%.
2. Influence of the type of fibre and type of fabric used in the reinforcement
  - Flexural tests
    ◦ The composites reinforced with sheep’s wool have a higher specific flexural strength and higher Young’s modulus than the ones reinforced with dog wool, with an average improvement of 17.44% and 50.93%, respectively;
    ◦ The composites reinforced with crocheted fabrics have the highest specific flexural strength and Young’s modulus values, with average values of 21.71% and 45.99%, respectively, compared to composites reinforced with woven fabrics, and average values of 137.57% and 186.68%, respectively, compared to composites reinforced with knitted fabrics.
  - Tensile tests
    ◦ Composites reinforced with sheep’s wool have a higher specific tensile strength and higher Young’s modulus than the ones reinforced with dog wool, with an average improvement of 33.16% and 95.06%, respectively;
    ◦ If the reinforcement is made of dog wool, the composites reinforced with crocheted fabrics have the highest specific tensile strength, but if the reinforcement is made of sheep’s wool, the composites reinforced with woven fabrics are the ones with the highest specific tensile strength;
    ◦ Composites reinforced with woven fabrics made from dog wool have the highest modulus, but in the composites where sheep’s wool was used, the ones reinforced with crocheted fabrics were the ones with the highest Young’s modulus values;
    ◦ Composites reinforced with sheep wool fibres exhibit higher strain at first failure/break values if the reinforcement is made by weaving, but if reinforcement is made by crochet or knitting, the composites reinforced with dog wool fibres exhibit the highest values;
    ◦ Composites reinforced with woven fabrics have the highest deformation at first break/failure values, with an average increase of 14.56% and 187.80% compared to composites reinforced with crocheted and knitted fabrics, respectively;
    ◦ Composites reinforced by woven fabrics are the ones with the highest toughness, with an average increase of 17.89% and 752.94% compared to composites reinforced with crocheted and knitted fabrics, respectively;
    ◦ If the reinforcement is made by weaving and crochet, the composites reinforced with sheep wool fibres have higher toughness values than those reinforced with dog wool fibres;

Additionally, from the tensile tests carried out on sheep’s and dog wool yarns, one can conclude that sheep’s wool is stiffer and can withstand more strain before breaking, while dog wool has higher strength and is able to absorb more energy before breaking.

Composites reinforced with natural fibres are gaining attraction across various sectors due to the environmental benefits and to the reduction in the manufacturing costs. Even though the biocomposites produced in this work cannot be used in structural applications, they can be used to produce interior and exterior vehicle components, such as door panels or seat pads. Also, they can be used in the automobile industry to reduce the overall weight and the production costs, and to improve fuel efficiency. In the construction industry, these composites can be used to manufacture windows, windows frames, doors, roof titles, and ceilings [35,36].

To optimize the mechanical properties, it is suggested that in future works the yarns made from the dog wool be spun mechanically. This could be an important point to evaluate, as the mechanical spinning and twisting of the yarns can affect the mechanical characteristics of the composites. Another interesting point to study is the use of fibres from different breeds of sheep and dogs, which would allow a more extensive analysis of the mechanical properties of the composites reinforced with animal fibres.

## Figures and Tables

**Table 1:** Properties of natural and synthetic fibres (adapted from [4,13,14]).

Fibre	Diameter (µm)	Length (mm)	Density (g/cm^3^)	Tensile Strength (MPa)	Specific Tensile Strength (MPa/g·cm^−3^)	Young’s Modulus (GPa)	Specific Young’s Modulus (GPa/g·cm^−3^)	Failure Strain (%)
Ramie	20	900–1200	1.5	400–938	270–620	44–128	29–85	2.0–3.8
Flax	12–16	5–900	1.5	345–1830	230–1220	27–80	18–53	1.2–3.2
Hemp	16–50	5–55	1.5	550–1110	370–740	58–70	39–47	1.6
Jute	17–20	1.5–120	1.3–1.5	393–800	300–610	10–55	7.1–39	1.5–1.8
Sisal	200–400	900	1.3–1.5	507–855	362–610	9.4–28	6.7–20	2.0–2.5
Alfa	-	350	1.4	188–308	134–220	18–25	13–18	1.5–2.4
Cotton	11–20	10–60	1.5–1.6	287–800	190–530	5.5–13	3.7–8.4	3.0–10
Coir	10–20	20–150	1.2	131–220	110–180	4–6	3.3–5	15–30
Silk	-	Continuous	1.3	100–1500	100–1500	5–25	4–20	15–60
Feather	-	10–30	0.9	100–203	112–226	3–10	3.3–11	6.9
Wool	16–40	38–152	1.3	50–315	38–242	2.3–5	1.8–3.8	13.2–35
E-Glass	-	Continuous	2.5	2000–3000	800–1400	70	29	0.5–3
S-Glass	-	-	2.5	4570	-	86	-	2.8
Aramid	-	-	1.4	3000–3150	-	63–70	-	2.5–3.7
Carbon	-	-	1.4	4000	-	23–240	-	1.4–1.8
Kevlar	-	-	1.44	3000	-	18–25	-	2.5–3.7

**Table 2:** Properties of sheep’s wool (adapted from [14,25]).

Property	Value
Density (g/cm^3^)	1.07–1.3
Length (mm)	25–355
Diameter (µm)	15–40
Young’s modulus (GPa)	2–5
Stretching (%)	25–50

**Table 3:** Properties of dog wool (adapted from [5]).

Property	Value
Density (g/cm^3^)	1.31–1.34
Length (mm)	32
Diameter (µm)	30–60
Young’s modulus (GPa)	2–3
Stretching (%)	43.7

**Table 4:** SR GreenPoxy 56 resin properties (adapted from [26]).

Property	Tensile	Flexural
Young’s modulus (GPa)	3.3	3.4
Maximum strength (MPa)	49	-
Strength (MPa)	48	114
Strain at max. load (%)	1.6	4.2
Failure strain (%)	1.6	5.5

**Table 5:** Fibre fraction of the composites produced.

Sample ID	Fibre Weight (g)	Composite Weight (g)	Fibre Fraction (%)
S_C_2Y_N3	42.48	121.68	34.91
S_C_2Y_N5	41.48	142.20	29.17
S_C_3Y_N5	61.20	172.23	35.53
D_C_1Y_N5	62.96	165.89	37.95
S_K_3Y_N5	33.16	157.12	21.10
D_K_1Y_N5	37.04	140.74	26.31
S_W_3Y	24.12	102.47	23.54
D_W_1Y	35.69	110.68	32.25

**Table 6:** Distance between supports during the flexural tests.

Sample ID	Distance Between Supports (mm)
S_C_2Y_N3	49
S_C_2Y_N5	64
S_C_3Y_N5	52
D_C_1Y_N5	55
S_K_3Y_N5	58
D_K_1Y_N5	66
S_W_3Y	32
D_W_1Y	40

**Table 7:** Composite volume fractions of matrix and fibres, and stiffness of matrix and fibres used in the rule of mixtures.

Sample ID	*V_m_*	*V_f_*	*E_m_* (GPa)	*E_f_* (GPa)
S_C_2Y_N3	0.3427	0.6573	3.3	0.27
S_C_2Y_N5	0.2859	0.7141	3.3	0.27
S_C_3Y_N5	0.3489	0.6511	3.3	0.27
D_C_1Y_N5	0.3472	0.6528	3.3	0.19
S_K_3Y_N5	0.2064	0.7936	3.3	0.27
D_K_1Y_N5	0.2370	0.7630	3.3	0.19
S_W_3Y	0.2304	0.7696	3.3	0.27
D_W_1Y	0.2927	0.7073	3.3	0.19

**Table 8:** Young’s modulus results obtained experimentally and estimated considering the isostress and isostrain conditions.

Sample ID	Isostress Condition (GPa)	Isostrain Condition (GPa)	Experimentally Obtained (GPa)
S_C_2Y_N3	0.68	22.62	1.04
S_C_2Y_N5	0.78	24.34	0.85
S_C_3Y_N5	0.67	22.43	2.39
D_C_1Y_N5	0.49	22.20	1.40
S_K_3Y_N5	1.00	26.75	1.96
D_K_1Y_N5	0.68	25.63	0.68
S_W_3Y	0.92	26.02	2.15
D_W_1Y	0.57	23.90	1.83

## Data Availability

Data are contained within the article.
